# Hypoalbuminemia in patients following their recovery from severe coronavirus disease 2019

**DOI:** 10.1002/jmv.27002

**Published:** 2021-05-03

**Authors:** Kameran M. Ali, Ayad M. Ali, Hassan M. Tawfeeq, Grazziela P. Figueredo, Hassan M. Rostam

**Affiliations:** ^1^ Medical Lab Technology Department, Kalar Technical Institute Sulaimani Polytechnic University Kalar Kurdistan Region Iraq; ^2^ Department of Chemistry, College of Sciences University of Garmian Kalar Kurdistan Region Iraq; ^3^ School of Computer Science University of Nottingham Nottingham UK; ^4^ Immunology & Immuno‐bioengineering Group, School of Life Sciences, Faculty of Medicine & Health Sciences University of Nottingham Nottingham UK; ^5^ Department of Medicine, College of Medicine University of Garmian Kalar Kurdistan Region Iraq

**Keywords:** COVID‐19, CRP, ESR, postrecovery, serum albumin

## Abstract

Coronavirus disease 2019 (COVID‐19) is caused by a contagious virus that has spread to more than 200 countries, territories, and regions. Thousands of studies to date have examined all aspects of this disease, yet little is known about the postrecovery status of patients, especially in the long term. Here, we examined erythrocyte sedimentation rate (ESR), C‐reactive protein (CRP), and serum albumin biomarkers in patients with a history of severe and mild‐to‐moderate COVID‐19 following their recovery. In patients with severe COVID‐19 serum albumin had a strong negative correlation with both ESR and CRP levels (*R* ^2^ = − 0.861 and *R* ^2^ = − 0.711), respectively. Also, there was a positive correlation between ESR and CRP level (*R* ^2^ = 0.85) in the same group. However, there was no correlation between these biomarkers among mild‐to‐moderate COVID‐19 patients. In addition, no correlation was recorded between the severe and mild‐to‐moderate COVID‐19 groups. This finding highlights the sustained elevation of ESR and CRP level and reduced serum albumin level that may persist postrecovery in patients with a history of severe COVID‐19.

## 1 INTRODUCTION

Beginning in late December 2019 in Wuhan, China,^1^ the ongoing coronavirus disease 2019 (COVID‐19) pandemic, which was caused by the spread of a novel coronavirus, severe acute respiratory syndrome coronavirus 2 (SARS‐CoV‐2)—named for the similarity of its symptoms to those induced by the SARS‐CoV‐2^2^—achieved worldwide reach within 3 months of its discovery.^3^, ^4^ Accordingly, on 11 March 2020, the World Health Organization labeled COVID‐19 as pandemic disease.^5^

Patients with COVID‐19 may show flu‐like symptoms such as fever, cough, dyspnea, myalgia, and fatigue. Those with serious forms of the disease can experience severe pneumonia, respiratory failure, multiorgan dysfunction, and death.^6^, ^7^ Gastrointestinal symptoms such as diarrhea, nausea, and vomiting have also been reported, along with a loss of the senses of taste and smell.^8^, ^9^ Since the start of the initial outbreak, scientists have made grade strides in understanding the pathophysiology and progression of this disease.^10^, ^11^, ^12^

The clinical manifestations of patients infected with SARS‐CoV‐2 can be stratified as mild, moderate, severe, and critical.^13^ The majority of affected patients (81%) suffer mild/moderate symptoms, whereas severe and critical cases total 14% and 5% of infected cases, respectively.^14^

Several biological markers have been found to correlate with the severity of COVID‐19, including high C‐reactive protein (CRP) level, high erythrocyte sedimentation rate (ESR), and low serum albumin level (hypoalbuminemia).^11^, ^15^, ^16^, ^17^ These biomarkers in parallel with clinical symptoms can be used to determine with greater confidence the likely progression and severity of the disease in a certain case.^18^ CRP is an exquisitely sensitive systemic marker for the acute phase response to inflammation, infection, and tissue damage^19^ and it has been reported that CRP levels are positively correlated with the severity of COVID‐19.^20^ Another study reported the observation of high ESR levels in patients suffering severe COVID‐19 symptoms relative to those with less severe disease due to an increase in the inflammation inherent in the former group.^15^ In addition, other studies have suggested the serum albumin level to be a vital indicator of status in patients with severe COVID‐19.^17^, ^21^

Since the appearance of COVID‐19 on the global stage, much research has been conducted regarding this disease.^10^, ^11^, ^12^, ^18^ To date, however, even though millions of people have recovered from this condition, limited follow‐up studies exist that have focused on the postrecovery health status of these individuals.^22^ Here, we examined the ESR, CRP levels, and serum albumin levels postrecovery in patients with a history of severe COVID‐19 and compared the collected values with the same parameters in a population with a history of mild‐to‐moderate COVID‐19.

## 2 MATERIALS AND METHODS

### 2.1 Real‐time reverse transcription polymerase chain reaction assay for SARS‐CoV‐2

A total of 46 hospitalized patients were included in this study. The diagnostic tests were performed for each patient, and pharyngeal swab samples collected for extracting 2019‐nCoV RNA. After collection, the total RNA was automatically extracted within 45 min using the Qiagen EZ1 Advanced XL system (Qiagen). Then, the presence of SARS‐CoV‐2 was detected by real‐time reverse transcription‐polymerase chain reaction (RT‐PCR) amplification of SARS‐CoV‐2 open reading frame 1ab (ORF1ab) and envelope (E) genes fragments using PowerChek SARS‐CoV‐2 Real‐Time PCR Kit (KogeneBiotech). Conditions for amplification were 50°C for 30 min, 95°C for 10 min, followed by 40 cycles of 95°C for 15 s and 60°C for 1 min. When two target genes (ORF1ab, E) tested positive by specific real‐time RT‐PCR, the case would be transferred to the laboratory for confirmation. A cycle threshold value (*C*_t_‐value) ≤ 36.7 was defined as a positive test, and the *C*_t_‐value of greater than 36.7 was defined as a negative test or recovered.

### 2.2 COVID‐19 severity category

The criteria for severity of COVID‐19 were defined according to the diagnosis and treatment protocol for novel coronavirus pneumonia (Version 7) as mild, moderate, and severe.^23^ Mild cases the patient shows mild clinical symptoms with no sign of pneumonia on imaging; moderate cases the patient shows fever and respiratory symptoms with radiological findings of pneumonia; severe cases have any of the following criteria, respiratory distress (≧30 breaths/min), oxygen saturation ≤93% at rest, arterial partial pressure of oxygen (PaO_2_)/fraction of inspired oxygen (FiO_2_) ≦ 300 mmHg (1 mmHg = 0.133 kPa).

Postrecovery, means the time period after recovery when the COVID‐19 patients discharged from the hospital and COVID‐19 signs and symptoms disappeared after the negative RT‐PCR^24^ that is, returning to a normal or healthy state after a period of COVID‐19 disease. Based on, blood samples were collected from recovered COVID‐19 patients within 2–4 weeks (with a mean of 20.6 ± 3.3 days) after their negative RT‐PCR.

Considering the above criteria, recovered patients were divided into two groups; 23 mild–moderate cases and 23 severe postrecovered COVID‐19 cases.

### 2.3 Biological marker test

Biological marker tests including CRP and serum albumin were assessed for mild, moderate, and severe groups using an automated multiparametric analyzer (Cobas c111; Roche Diagnostics) and ESR were tested by the Westergren method.^25^

### 2.4 Ethics declarations

All methods were carried out in accordance with the relevant guidelines and regulations. Also, we confirm that all experimental protocols were approved by the Ethics Licensing Committee of the Kalar Technical Institute at the Sulaimani Polytechnic University Committee (No. 01 on August 1, 2020). In addition, informed consent was obtained from all subjects or if subjects are under 18, from a parent and/or legal guardian.

### 2.5 Statistical analysis

Pearson correlation and polynomial regressions were employed to understand the relationship between ESR, CRP, and serum albumin biomarkers between mild, moderate, and severe postrecovery COVID‐19 patients. Also, an unpaired *T*‐test has been used to study differences of body weight loss between both groups.

## 3 RESULTS AND DISCUSSION

Patients infected with SARS‐CoV‐2 can be assessed clinically by using quantitative measurements of numerous biomarkers such as ESR, CRP level, and serum albumin level. Monitoring of those biomarkers could play a key role in reviewing the pathological development and suggesting the prognosis and outcomes of the disease.^26^

In this study, all cases were identified by RT‐PCR and categorized into two groups (mild‐to‐moderate and severe) according to the status of their disease. ESR, CRP levels, and serum albumin levels were measured in both groups and we found that albumin had a strong negative correlation with ESR (*R* ^2^ = − 0.861) and CRP level (*R* ^2^ = − 0.711) postrecovery in patients with a history of severe COVID‐19 (Table 1).

**TABLE 1:** Correlation analysis between ESR, CRP, and albumin

	ESR severe	CRP severe	Serum albumin severe	ESR mild–moderate	CRP mild–moderate	Serum albumin mild–moderate
ESR severe	1.0000	0.8534	−0.8610	0.1206	0.0868	−0.3505
CRP severe	0.8534	1.0000	−0.7114	−0.0487	−0.0457	−0.3242
Serum albumin severe	−0.8610	−0.7114	1.0000	0.0784	0.1265	0.2419
ESR mild–moderate	0.1206	−0.0487	0.0784	1.0000	0.6149	−0.0504
CRP mild–moderate	0.0868	−0.0457	0.1265	0.6149	1.0000	−0.3277
Serum albumin mild–moderate	−0.3505	−0.3242	0.2419	−0.0504	−0.3277	1.0000

*Note*: In severe COVID‐19 postrecovery group and mild/moderate COVID‐19 postrecovery group. Also, correlation analysis between severe and mild/moderate COVID‐19 postrecovery groups, *n* = 46 patients.

Abbreviations: COVID‐19, coronavirus disease 2019; CRP, C‐reactive protein; ESR, erythrocyte sedimentation rate.

Hypoalbuminemia is seen more predominantly in severe COVID‐19 cases than mild cases.^27^ However, no study has yet evaluated the levels of albumin nor the effect of such on the health of patients with a history of COVID‐19 after their recovery. In our study, we observed persistent hypoalbuminemia postrecovery in patients with a history of severe COVID‐19. Although the mechanisms for hypoalbuminemia in COVID‐19 have not been studied thoroughly,^28^ albumin is considered a major serum protein produced by hepatic cells,^28^, ^29^ and has a critical role in human health. As such, hypoalbuminemia is considered a sinister clinical sign in COVID‐19 viral infection that may be attributed to the release of major acute phase cytokines into the blood vessels during cytokine storm^17^ or due to an increase in vascular permeability, which allows the albumin to diffuse into the extravascular space.^30^ A reduction in albumin synthesis may also be the result of anorexia caused by SARS‐CoV‐2 viral infection.^17^ Thus, a high protein nutrition and eventual albumin administration to the COVID‐19 patients should be considered.

In the present study, we found that ESR increased in all severe COVID‐19 postrecovery patients. Similar results were found by Pu et al.^31^ who observed an elevated level of ESR in a case study of a patient recovered from severe COVID‐19 infection. This finding may justify the strong negative correlation between ESR and albumin because albumin retards the sedimentation of erythrocytes and decreases the rouleaux formation while hypoalbuminemia accelerates it,^32^ in contrary to other plasma proteins in which high level of them speed it up.^33^

Our study revealed a high concentration of CRP in severe COVID‐19 postrecovery patients. A significant negative correlation was also found between albumin level and the inflammatory indicator, CRP (*R* ^2^ = − 711) (Table 1). Studies have determined a significant increase of CRP concentration in severe COVID‐19 patients.^34^, ^35^, ^36^ However, our data are the first study related to the COVID‐19 postrecovery patients. CRP is produced by the liver as a nonspecific immune protein and it is considered as a signal of systemic inflammation^37^ CRP level in serum also can be affected with the level of other serum proteins which are produced by liver cells.^38^, ^39^

Ponti et al.^40^ found the severity of COVID‐19 is positively correlated with ESR and CRP, while no study on both biomarkers after the recovery of COVID‐19 patients has been recorded yet. Our data has revealed a positive correlation between ESR and CRP (*R* ^2^ = 0.85) in severe COVID‐19 postrecovery patients. Elevation of inflammatory biomarkers can be considered as a parameter for COVID‐19 infection and its severity.^26^

In the mild–moderate COVID‐19 postrecovery group, our data showed nonsignificant positive correlation between ESR and CRP (0.6149). In addition, in the same group there was neither correlation between CRP and albumin (–0.3277) nor between ESR and albumin (−0.0504; Table 1). Several studies have revealed low ESR and CRP in mild–moderate COVID‐19 patients,^40^, ^41^, ^42^ while other studies showed a slight decrease of serum albumin in the same group when compared with severe cases.^41^, ^43^ Most importantly no studies conducted on postrecovery patients addressing changes in those markers yet. In mild–moderate COVID‐19 patients the inflammatory proteins that have an effect on ESR boosting maintain in their minimum level, subsequently ESR stays in their normal range.^44^ Our study seems to be one of the first attempts to observe those biological markers in COVID‐19 postrecovery patients.

In Table 1, data analyses showed no correlation between mild, moderate, and severe groups in COVID‐19 postrecovery patients when both groups compared each other in terms of ESR, CRP, and serum albumin markers.

A study by Kermali et al.^26^ showed a significant difference in ESR, CRP, and serum albumin between mild and severe COVID‐19 patients. However, the difference between moderate and severe groups was observed only in ESR.

**FIGURE 1:**
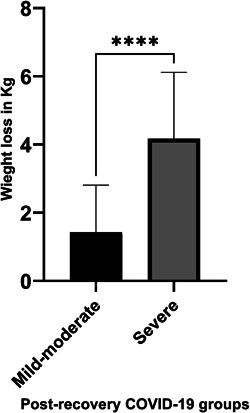
The difference in losing bodyweight in kilograms between severe mild–moderate coronavirus disease 2019 (COVID‐19) postrecovery group (black), and severe COVID‐19 postrecovery group (gray)

The present study showed a significant difference in the body weight loss between mild, moderate, and severe postrecovery COVID‐19 patient groups (*p* < 0.0005), body weight loss average for mild–moderate group was 1.43 ± 1.38, while for the severe group was 4.17 ± 1.95 (Figure 1). Several factors may contribute to body weight loss and malnutrition in COVID‐19 patients,^45^, ^46^ such as; systemic inflammation, high CRP level,^46^ hypoalbuminemia, inadequate protein, and caloric intake. Also, inflammation induces anorexia, reduces the effective use of dietary protein and energy intake, and augments catabolism of the key somatic protein, albumin which has consequences on body weight loss.^47^

In conclusion, we found a prolonged increase of ESR, CRP, and decrease of serum albumin in severe COVID‐19 postrecovery patients. We also discovered a strong negative correlation of albumin with both ESR and CRP in the group. Therefore, further study on albumin administration and ESR/CRP de‐escalation is recommended which helps COVID‐19 postrecovery patients to avoid further consequences of the disease.

## CONFLICT OF INTERESTS

The authors declare that there are no conflict of interests.

## AUTHOR CONTRIBUTIONS

Kameran M. Ali and Ayad M. Ali have performed lab work. Hassan M. Tawfeeq and Hassan M. Rostam have contributed in the writing. Grazziela Figueredo has analyzed the data.
